# Supplementary material for: Glutathione Plays a Positive Role in the Proliferation of Pinus koraiensis Embryogenic Cells
Source: Int J Mol Sci. 2022 Nov 24;23(23):14679. doi: 10.3390/ijms232314679 (PMC9736457; doi:10.3390/ijms232314679)
Supplement: Supplementary file 1 [file ijms-23-14679-s001.zip › ijms-1983656-supplementary.pdf]

International Journal of *Molecular Sciences*

MDPI

Supplementary Material

Supplementary Table S1. qRT-PCR primer sequences in Korean pine

Unigenes	Forward primer	Reverse primer
c154707.graph_c1（Internal reference）	CAGCTCAGCTTGAAATGA	ACGAGTACTGTGAGGAAA
c180344.graph_c0	CGACATGGTTCCAAACTA	GACCCTGAGAAAATTGTTG
c133544.graph_c0	AGGGGATGAATTCAGCATA	CGCATTCACATACTTTCGA
c163132.graph_c0	GACACACAATCCCAATTG	GGTAGTTTGTGTTTGCAG
c187964.graph_c1	TGGAAACCCTGTTTGTGAA	GCAATGGCTCGGTCATAT
c188830.graph_c0	CTCCTAGCTCTCAGGTCAT	CTTTGCGATGAGTTGTGC
c181305.graph_c0	CTGGTTGGTTACACGTTCC	TGTGCATGGATAGACCGAT
c187768.graph_c0	ATGGACATGAAGCATTGC	CGGTGATTGGTTACAACC
c183265.graph_c0	TTCTCAATCCGAATCTGGAA	AGGAGATGTCACCGTAGTA
c187512.graph_c0	TCTCCGATCTTCAGGAATCA	TGGCAACCATCAATCAAGT
c187027.graph_c1	TGATGAAGGCTTCCACAGA	CAAGTGCTATGCGAACCC
c187045.graph_c0	GCAAGAGCTACAGATCAA	GGACGAAATCGTATTCAAC

Supplementary Table S2. Different genes related to cytokinin between F and S cell lines of Korean pine

Gene ID	Gene family	Functional description	Regulated
c185059.graph_c0	AHK2_3_4	Arabidopsis histidine kinase 2/3/4 (cytokinin receptor)	down
c189912.graph_c0	ARR-B	Two-component response regulator ARR-B family	up

Supplementary Table S3. Functional description of qRT-PCR validated genes in Korean pine

Gene ID	Gene family	Functional description
c133544.graph_c0	GST	Glutathione S-transferase
c180344.graph_c0	GST	Glutathione S-transferase
c181305.graph_c0	GST	Glutathione S-transferase
c183265.graph_c0	DHAR	Glutathione dehydrogenase/transferase
c187027.graph_c1	GST	Glutathione S-transferase
c187045.graph_c0	GST	Glutathione S-transferase
c187964.graph_c1	GST	Glutathione S-transferase
c188830.graph_c0	GST	Glutathione S-transferase
c190620.graph_c2	ODC1	Ornithine decarboxylase
c187768.graph_c0	GULO	L-Gulonolactone oxidase
c163132.graph_c0	SAUR	Auxin-responsive protein SAUR71-like
c187512.graph_c0	ARF	Auxin response factor 2-like isoform X1

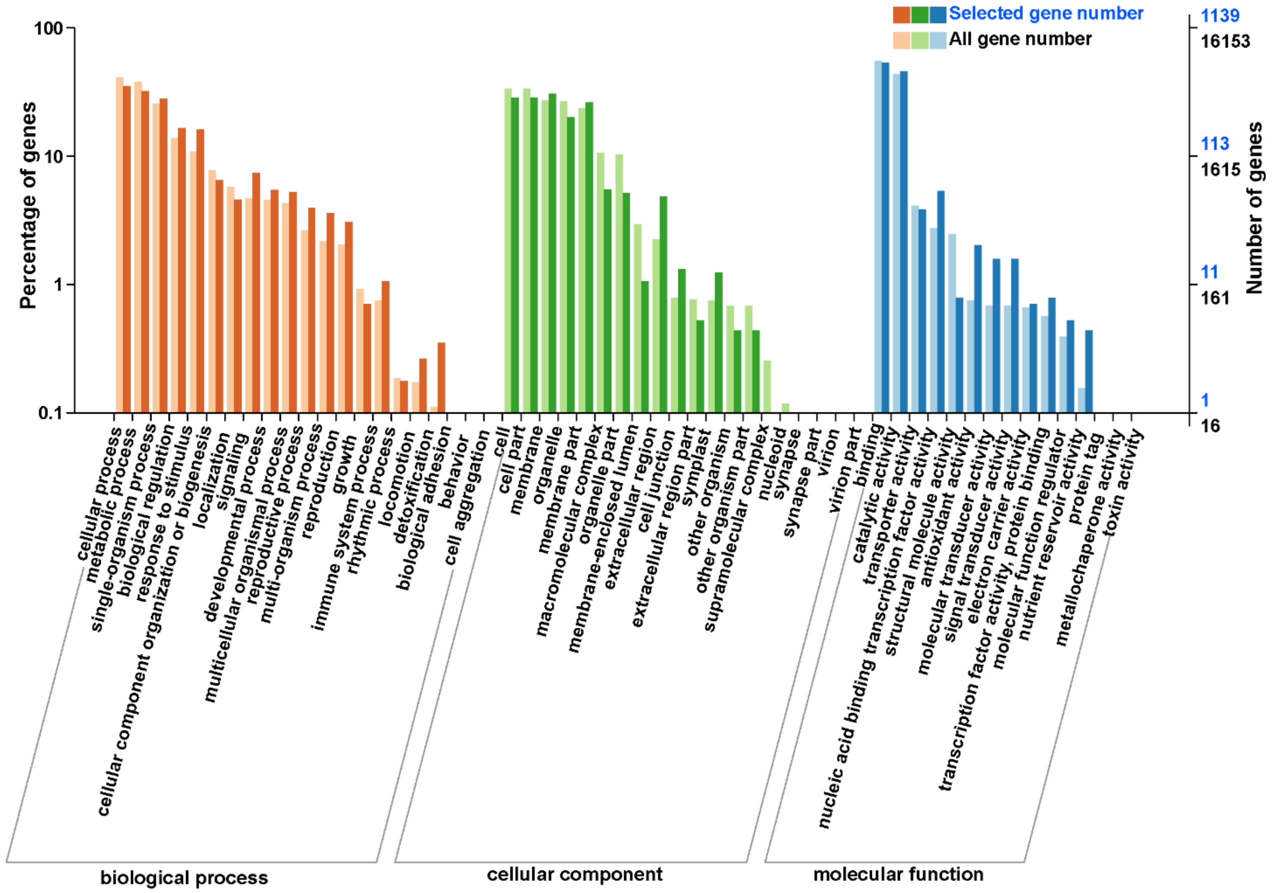

Supplementary Figure S1. Annotation statistics diagram of the GO secondary node of the differentially expressed genes of F and S cell lines of Korean pine.

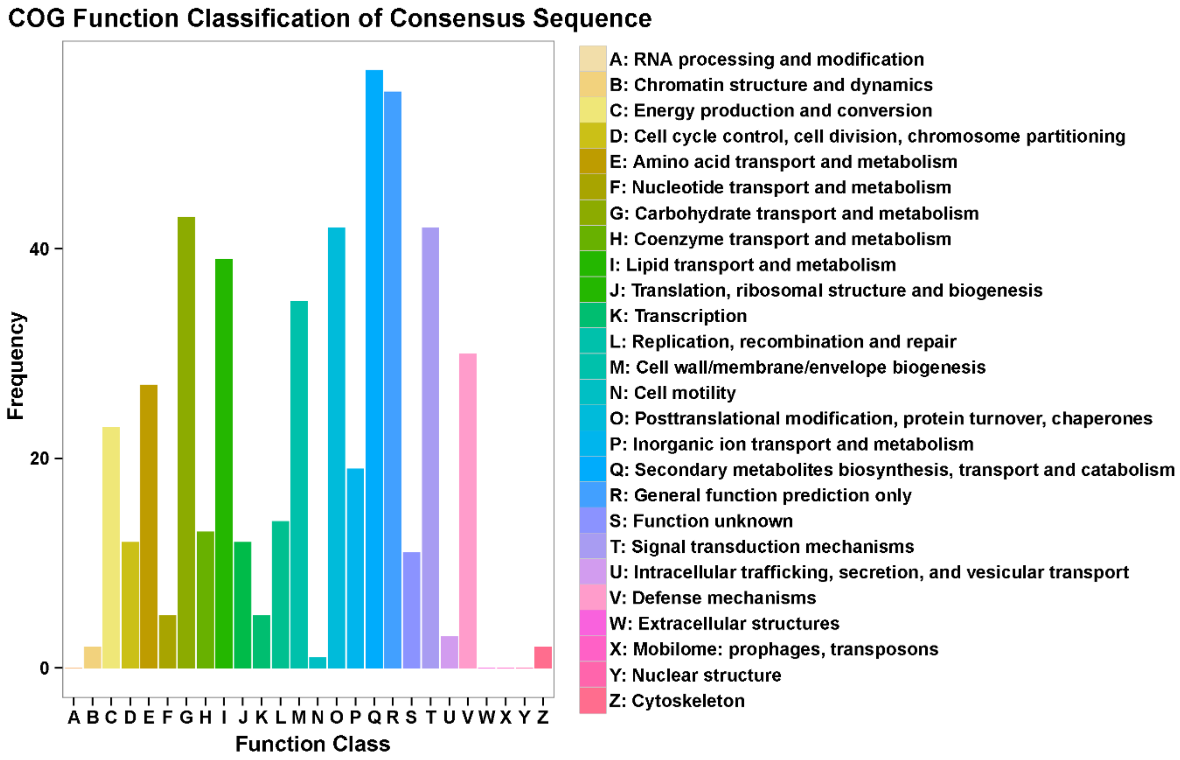

Supplementary Figure S2. Classification statistics of COG annotation of differentially expressed genes in F and S cell lines of Korean pine.

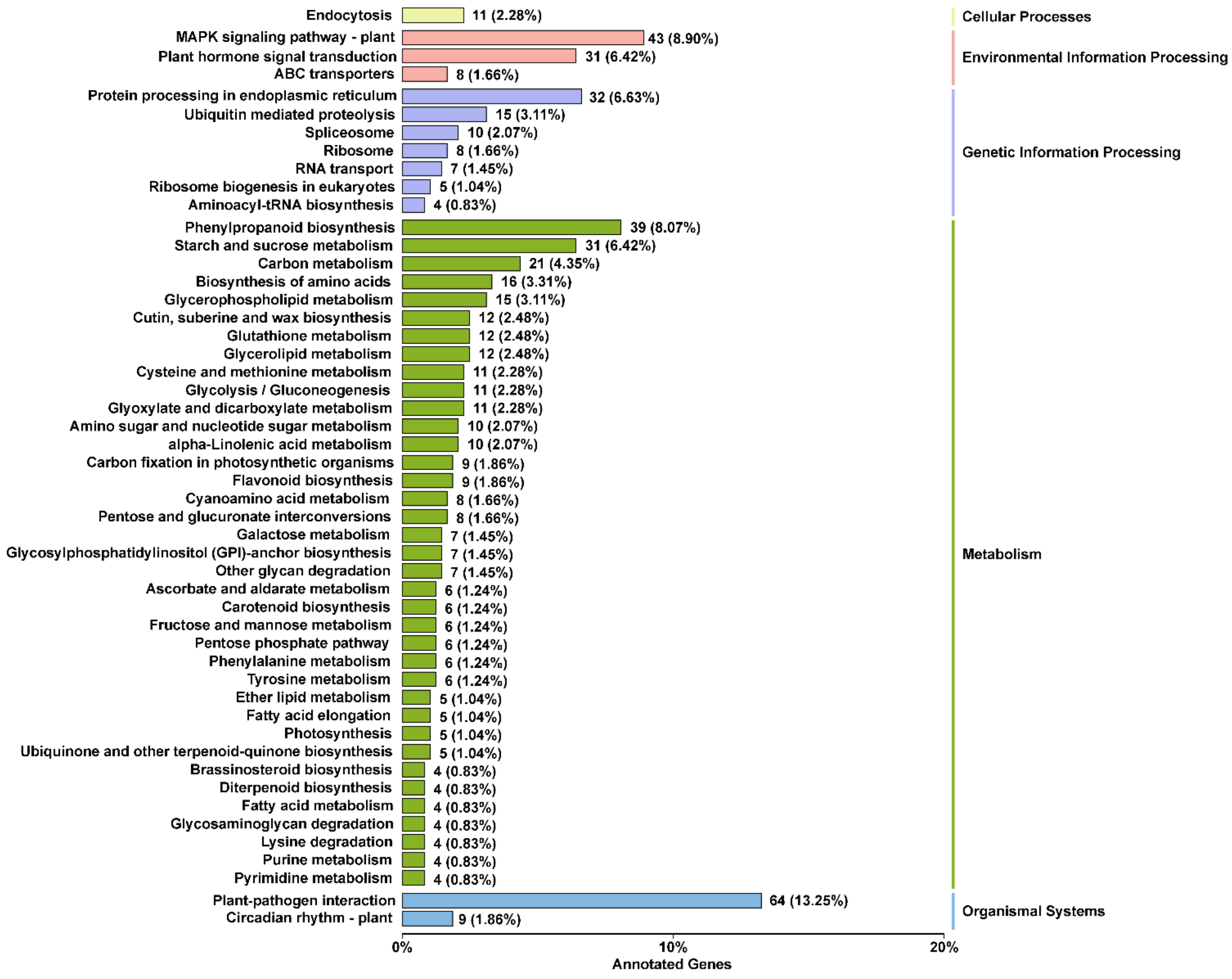

Supplementary Figure S3. KEGG Pathway classification of differentially expressed gene in two cell lines of Korean pine.
